# *Bifidobacterium* mechanisms of immune modulation and tolerance

**DOI:** 10.1080/19490976.2023.2291164

**Published:** 2023-12-06

**Authors:** Samuel J Gavzy, Allison Kensiski, Zachariah L Lee, Emmanuel F Mongodin, Bing Ma, Jonathan S Bromberg

**Affiliations:** a Center for Vascular and Inflammatory Diseases, University of Maryland School of Medicine, Baltimore, MD, USA; b Department of Surgery, University of Maryland School of Medicine, Baltimore, MD, USA; c Institute for Genome Sciences, University of Maryland School of Medicine, Baltimore, MD, USA; d Department of Microbiology and Immunology, University of Maryland School of Medicine, Baltimore, MD, USA

**Keywords:** Bifidobacteria, gut microbiome, immune homeostasis, metabolites, tolerogenic immune responses, live biotherapeutics

## Abstract

*Bifidobacterium* is a widely distributed commensal bacterial genus that displays beneficial pro-homeostatic and anti-inflammatory immunomodulatory properties. Depletion or absence of *Bifidobacterium* in humans and model organisms is associated with autoimmune responses and impaired immune homeostasis. At the cellular level, *Bifidobacterium* upregulates suppressive regulatory T cells, maintains intestinal barrier function, modulates dendritic cell and macrophage activity, and dampens intestinal Th2 and Th17 programs. While there has been a large volume of literature characterizing the probiotic properties of various *Bifidobacterial* species, the likely multifactorial mechanisms underlying these effects remain elusive, in particular, its immune tolerogenic effect. However, recent work has shed light on *Bifidobacterium* surface structural polysaccharide and protein elements, as well as its metabolic products, as commensal mediators of immune homeostasis. This review aims to discuss several mechanisms *Bifidobacterium* utilizes for immune modulation as well as their indirect impact on the regulation of gut microbiome structure and function, from structural molecules to produced metabolites. These mechanisms are pertinent to an increasingly networked understanding of immune tolerance and homeostasis in health and disease.

## PART I. Overview of *Bifidobacterium* immune modulatory abilities

*Bifidobacterium* is a gram-positive, anaerobic, saccharoclastic, non-motile commensal bacterial genus. It contains 10 phylogenetic clusters and has a broad host range within the mammalian gastrointestinal tract.^1,2^ *In vitro* culture of Bifidobacteria require highly specific conditions given their requirement for an anaerobic environment and metabolic precussors.^3,4^ *Bifidobacterium* exhibits genomic differences that partly reflect the gene acquisition events required to thrive in different host ecological niches. *Bifidobacterium* tropism and niche adaption underpin its diverse role in host immune regulation.

*Bifidobacterium* has been implicated as an immunomodulator or biomarker of human disease, serving as both a driver and protector. Various strains are commonly used as live biotherapeutics^5^ and demonstrate beneficial immunomodulatory and anti-inflammatory properties. These include upregulation of suppressive Foxp3+ regulatory T cells (Tregs),^6^ improvement of intestinal barrier function,^7^ and dampening of intestinal Th2 and Th17 programs.^8^ On the other hand, absence or reduction of *Bifidobacterium* species has been implicated in multiple autoimmune and autoinflammatory conditions in humans. For instance, decreased gut levels of several *Bifidobacterium* species in humans are associated with the microbiome fingerprint of treatment-naïve Crohn’s disease (CD).^9^ Decreased levels of gut *Bifidobacterium infantis* are correlated with Guillain-Barré Syndrome in humans.^10^ Mice treated with *B. pseudolongum* ATCC25526 and receiving allogeneic heterotopic heart transplants with long-term immunosuppression display improved long-term graft survival and decreased allograft inflammation.^11^ Contrary to its classical protective role, enrichment of *Bifidobacterium* is also associated with Parkinson’s disease in a meta-analysis of patient gut microbiome data.^12^ Similarly, *Bifidobacterium* is enriched in the stool of patients with ulcerative colitis (UC) compared to healthy controls.^13^ These results suggest that Bifidobacteria possess both multifactorial immunomodulatory mechanisms and diverse strain-mediated immune effects.

Bifidobacteria also modulate immune responses at the level of the gut mucosa. In mice treated with *Bifidobacterium adolescentis* ATCC15703, the levels of pro-inflammatory cytokines TNFα, IL-6, IL- 1β, IL-18, IL-22, and IL-9 in colon homogenates are lower than controls,^14^ while anti-inflammatory IL-10 and Th2-type cytokines IL-4 and IL-5 are higher. Tregs are also increased in the colons of colitic mice receiving *B. adolescentis* ATCC15703. Similarly, germ-free mice colonized with *Bifidobacterium bifidum* strain PRI1 have increased Tregs in the colonic lamina propria.^6^ This effect is facilitated by colon lamina propria dendritic cells (DCs), which have increased mRNA expression of IL-10, GM-CSF, TGFβ1, Indoleamine 2,3-dioxygenase, PTGS2, and PD-1, as well as the co-stimulatory molecules CD86 and CD40 after treatment with PRI1. *In vitro* treatment of DCs with PRI1 followed by co-culture with naïve CD4 T cells leads to enhanced Treg induction and IL-10 production.^6^

*Bifidobacterium* immunomodulatory programs vary in phenotype and intensity at the species and strain levels, including pro-inflammatory effects.^15^ The immunomodulatory effect of *Bifidobacterium* was shown to be independent of its phylogeny.^16^ Bone marrow-derived DCs co-cultured with *Bifidobacterium animalis* subsp. *lactis* 5764 (Bl 5764) display high levels of maturation and costimulatory molecules CD40, CD86, and MHC II in comparison to non-stimulated cells. When pre-treated DCs are co-cultured with naïve CD4 T cells, there is increased pro-inflammatory IL-17A and IL-17F production compared to control and groups treated with different bacterial species.^17^ Co-culture of the THP-1 human monocyte cell line with *Bifidobacterium breve* strains UCC2003 and JCM7017 results in MyD88-dependent NFκB and TNFα expression compared to untreated controls.^18^ Murine bone marrow derived macrophages express increased TNFα after co-culture with these *B. breve* strains, favoring an anti-inflammatory phenotype. While this co-culture model provides a controlled environment to investigate potential interactions between *Bifidobacterium* and immune cells, the findings require careful interpretation and further validation using more sophisticated *ex vivo* or *in vivo* models. Strain-specific immunomodulation is likely rooted in niche adaption due to the different mammalian gut microenvironments from which each strain has been isolated. Thus, it is important to understand the properties of specific *Bifidobacterium* strains when planning for therapeutic application.

## PART II. *Bifidobacterium* modulates immune responses by affecting gut microbiome

Microbial species interact through the metabolites they consume and secrete, a process known as cross-feeding. Bifidobacteria play an essential role in producing metabolites utilized by other genera and even among different species and strains of *Bifidobacterium*.^19–26^ For example, *Anaerostipes caccae* L1–92 is an important butyrate producer that relies on metabolites produced by *Bifidobacterium* in order to establish itself in the infant gut. *A. caccae* L1–92 is unable to grow in monoculture, but co-culture with *B. infantis* ATCC15697 enables *A. caccae* L1–92 growth, which utilizes glucose and galactose along with the acetate generated by *B. infantis* ATCC15697 to produce butyrate.^20^ *Faecalibacterium prausnitzii* S3/L3 or A2–165 and *B. adolescentis* L2–32;*F. prausnitzii* A2–165 or ATCC 27,768 and *B. catenulatum* KCTC 3221; as well as *Eubacterium rectale*, ATCC 33,656, and *B. longum* NCC2705 share similar cross-feeding relationships. In these co-cultures, *Bifidobacterium* is necessary for either the establishment of a butyrate producer or for enhancing butyrate production through the metabolism of human milk oligosaccharides (HMOs) into monosaccharides and the production of acetate.^19,21,22^

This butyrogenic effect also results from the cross-feeding interaction between *Bifidobacteria* and *Clostridiales*, which negatively correlates with inflammatory bowel disorders.^19,21,27–29^ Short-chain fatty acids (SCFAs), particularly butyrate, are important for colonic Treg homeostasis.^30,31^ Singh *et al*. demonstrated the role of butyrate and niacin in the suppression of colonic inflammation and carcinogenesis through the activation of Gpr109a. This stimulated DCs and macrophages to produce IL-10, leading to enhanced differentiation of Tregs.^32^ Furthermore, co-culture of *F. prausnitzii* A2–165 or ATCC 27,768 and *B. catenulatum* KCTC 3221 results in the reduction of pro-inflammatory cytokines produced by HT-29 human colorectal adenocarcinoma cells and RAW 264.7 murine macrophages *in vitro* as well as decreased IL-8 in the colons of colitic mouse models.^22^ These studies indicate the important role of *Bifidobacterium* metabolism in modulating immune homeostasis directly or via cross-feeding. Furthermore, *Bifidobacterium* does not possess polyamine biosynthetic machinery for putrescene, a known immunomodulator, but administration of *B. animalis* subsp. *lactis* LKM512 increases the concentration of luminal putrescene by sufficiently acidifying the intestine and allowing for activation of polyamine biosynthesis by endogenous gut microbiota.^33^

From a cooperativity standpoint, *Bifidobacteria* can also negatively regulate the presence of pro-inflammatory metabolites such as trimethylamine N-oxide (TMAO), derived from gut microbiota-produced choline, which is associated with the development of atherosclerosis.^34^ Supplementation with *B. breve* Bb4 as well *as B. longum* BL1 and BL7 decreases plasma TMAO levels in mice.^35^ These pathways exemplify the complex interactions between different *Bifidobacterium* species and the host intestinal environment, including available carbohydrate nutrient sources and other members of the colonic microbiota, and their subsequent downstream effects on host immune responses.

Cross-feeding relationships also exist among various species of *Bifidobacterium* (Table 1), which subsequently affect metabolic production and immune properties. Different *Bifidobacterium* strains within the same species play different cross-feeding roles as well. *B. breve* UCC200 lacks the enzymes necessary for mucin degradation. Yet it proliferates to a greater extent in mucin-based media, similar to the human colon, when co-cultured with mucin-degrading *B. bifidum* PRL2010.^23^ Isolated *B. pseudocatelatum* strains LH9, LH11, LH13, LH14 that express fucosidase support the growth of *B. longum* strain LH12, which cannot degrade fucosylated HMOs.^24^ *B. bifidum* PRL2010 cannot grow in monoculture with xylan or starch-supplemented media, but grows in the same media when co-cultured with *B. adolescentis* 22 L, *B. breve* 12 L, or *B. thermophilum* JCM 1207.^25^ When co-cultured, *B. magnum* and *B. cuniculi* proliferate in starch-supplemented media, whereas co-culture with xylan results in *B. magnum* modulation of xylan-degrading genes, which supports *B. cuniculi*.^26^ This mutualism highlights the intricate relationships between various bacterial species in the gut microbiome and demonstrates that these direct and indirect interactions are all critical for determining the overall environment for immune stimulation versus homeostasis. The diverse applications of *Bifidobacterium* as a live biotherapeutic are contingent upon an array of strain-specific traits as well as interactions within the host milieu. This encompasses the interplay between host and gut microbiota, metabolic functionalities, adherence to intestinal epithelial cells, resilience to gastric acids and bile, immunomodulatory capacity, and competitive antagonism with pathogenic bacteria. Consequently, the functional dynamics of individual *Bifidobacterium* strains emerge from a multifaceted interplay of these distinct attributes.

**Table 1.** Cross-feeding relationships between *Bifidobacterium* and other microbiota species.

Strain	Metabolite/process	Beneficiary	Effect of cross-feeding	References
*B. infantis* ATCC15697	Acetate Human milk monosaccharides Lactate	*Anaerostipes caccae* L1–92	Butyrate production	20
*B. adolescentis* L2–32	Acetate	*Faecalibacterium prausnitzii* S3/L3 *F. prausnitzii* A2–165	Butyrate production	21
*B. catenulatum* KCTC 3221	Acetate	*F. prausnitzii* ATCC 27,768 *F. prausnitzii* A2–165	Butyrate production	22
*B. longum* subsp. *longum* NCC2705	Acetate	*Eubacterium rectale* ATCC 33,656	Acetate converting butyrate production	19
*Eubacterium rectale* ATCC 33,656	Xylose	*B. longum* subsp. *longum* NCC2705	Increased acetate production	19
*B. animalis* subsp. *lactis* LKM512	Acidifying intestine through lactate and acetate production	Endogenous gut microbiota represented by *Escherichia coli* (SK930) and *Enterococcus faecalis* (V583)	Polyamine biosynthesis (putrescine production)	33
*B. breve* UCC2003	Carbohydrates released from mucin degradation	*B. bifidum* PRL2010	Enhanced survival/growth	23
*B. pseudocatenulatum* LH9, LH11, LH13, LH14	Substrates released from fucosylated HMO degradation (acetate, ethanol, formate, and 1,2 propandiol)	*B. longum* LH12	Enhanced survival/growth	24
*B. longum* LH206	Substrates released from fucosylated HMO degradation (acetate, formate, ethanol, galactose, fucose, lactose)	*B. pseudocatenulatum* LH663 or LH657	Enhanced survival/growth	24
*B. breve* 12L; *B. adolescentis* 22L *B. thermophilum* JCM1207	Sugars released from either starch and/or xylan degradation	*B. bifidum* PRL2010	Enhanced survival/growth	25
*B. magnum*	Cooperation of extracellular amylases degrade starch	*B. cuniculi*	Enhanced survival/growth	26
*B. magnum*	Modulation of gene expression to support growth of *B. cuniculi*	*B. cuniculi*	*B. cuniculi* degrades xylan	26

## PART III. *Bifidobacterium* promotes a tolerogenic environment in various disease states

### Solid organ transplant

Given its role as a marker and inducer of anti-inflammatory and pro-tolerogenic immune effects, *Bifidobacterium* has been studied in solid organ transplant models. Mice receiving allogeneic heart transplants and *Bifidobacterium pseudolongum* ATCC25526 gavage along with daily tacrolimus immunosuppression display improved long-term allograft survival and decreased graft inflammation.^36^ Furthermore, treatment with *B. pseudolongum* ATCC25526 results in lymph node (LN) architectural changes, increasing the ratio between extracellular matrix glycoproteins laminin α4 and laminin α5, which is associated with tolerance.^37^ Treatment with *Bifidobacteria* also promotes intestinal homeostasis following transplantation. In a rat model of liver transplant, prolonged antibiotic use and semi-starvation for 4–5 weeks (associated with decreased ileocecal *Bifidobacterium*), and supplementation with *Bifidobacterium* and *Lactobacillus*-containing probiotic promotes partial restoration of intestinal microflora and improved intestinal barrier function.^38^ In a separate rat model of liver ischemia reperfusion injury, treatment with *Bifidobacterium catenulatum* ZYB0401 decreases serum TNFα and liver malondialdehyde and increases liver superoxide dismutase, which is associated with reduced liver injury.^39^ Rats administered *B. longum*-containing probiotic cocktail followed by liver transplant without immunosuppression display increased intestinal Treg cells and TGFβ in the serum and liver, with concomitant decreases in CD4/CD8 T cell ratios and serum IL-2.^40^

Exemplifying the dysbiotic state associated with immunosuppressant treatment, metagenomic analyses of human liver transplant recipients reveal deficits in beneficial *Bifidobacteriaceae* compared to healthy controls.^41,42^ Similarly, in renal transplant recipients, the gut microbiome displays a decreased abundance of multiple *Bifidobacterium* species^43^. However, the presence of the family *Bifidobacteriaceae* in the gut microbiomes of liver transplant recipients is associated with acute cellular rejection.^44^ As the family includes a large variety of species and strains that can have distinct immune modulatory properties, it is important to have species- and even strain-level taxonomic resolution to assist in characterizing the immunomodulatory effects of specific microbiota species and strains (Table 2).

**Table 2.** *Bifidobacterium* association with disease states and models.

Disease/Model	Strain/Genus	Presence (+) or Absence of (-) Bacteria Biomolecular/Cellular Effects	Host	References
Heart Transplant	*B. pseudolongum* ATCC25526	(+) ↑ LN laminin ratios; ↓ allograft inflammation and fibrosis	Mouse	36
Liver Transplant	*Bifidobacterium*	(+) ↑ intestinal barrier function	Rat	38
Kidney/Liver Transplant	*Bifidobacteriaceae/Bifidobacterium*	(-) depleted in transplant recipients	Human	41–43
Liver Transplant	*Bifidobacteriaceae*	(+) acute cellular rejection	Human	44
DSS Colitis	*B. pseudocatenulatum* MY40C and CCFM680, *B. infantis* CGMCC0460.1, *B. animalis* subsp. *lactis* XLTG11, *B. breve* M1 and M2	(+) ↓ colitis; ↑ tight junction and adherens junction proteins (β-catenin, claudin-3, occludin, and ZO-1) and ↑ mucin 2, IL-10 and PPARγ; ↓ TNFα and IL-6, downregulation of TLR4/NFκB pathway, production of colonic conjugated linoleic acid	Mouse	45–48
CTLA-4 blockade-induced colitis	*B. breve*	(+) ↓ colitis, ↑ Treg function	Mouse	49
Gliadin-Induced Enteropathy	*B. longum* CECT 7347; *B. longum* CECT 7347 co-administration with gliadin	(+) ↓ inflammatory cytokines and CD4 T cell mediated immune responses; restores intestinal structure; restores NFκB and IL-10, ↑ TNFα; ↓ overall CD4 and Treg, ↑ CD8 T cells; ↑ stress and intestinal absorption proteins, ↓ cellular homeostasis proteins (cytoskeletal organization, protein transport, gene transcription, retinoic acid binding, and cell starvation)	Mouse	50,51
Gliadin-Induced Enteropathy	*Bifidobacterium longum* NCC2705	(+) ↓ intraepithelial lymphocytosis	Mouse	52
Gliadin-Induced Enteropathy	*B. longum* ES1 and *B. bifidum* ES2	(+) ↓ inflammatory cytokines	*In vitro*	53
Gliadin-Induced Enteropathy	*B. lactis*	(+)↓ gliadin-induced epithelial permeability; ↓ membrane ruffle formation; protected tight junctions	*In vitro*	54
EAMG	*B. animalis* subsp. Lactis BB12, *B. animalis* subsp. Lactis LMG S-28195	(+) ↓ thymic IFNγ, TNFα, IL-6, and IL-17	Rat	55
EAMG	*B. bifidum* with IRT5 probiotic cocktail	(+) ↑ IL-10, TGFβ, arginase 1, and aldh1a2 expression in DCs	Rat	56
EAE	*B. animalis* PTCC 1631 with *Lactobacillus plantarum* A7; *B. bifidum* with IRT5 probiotic cocktail	(+) ↓ disease progression; ↓ central nervous system inflammation; ↑ Treg in PLN and spleen; ↓ Th1 and Th17 via ↓ IL-17, IL-6, and IFNγ and ↑ TGFβ, IL-4, and IL-10	Mouse	57,58
Multiple Sclerosis	*Bifidobacterium*	(+) associated with disease	Human (adult and pediatric)	59,60
Shrimp tropomyosin-induced allergy	*B. lactis*	(+) ↑ Treg/Th17 ratios	Mouse	61
Shrimp tropomyosin-induced allergy	*B. infantis* 14.518	(+) ↑ DC maturation and accumulation of CD103+ tolerogenic DCs in Peyer’s patches and MLN; ↑ Tregs and ↓ Th2 responses	Mouse	62
Ova-induced food allergy	*B. breve M*-16V	(+) ↓ Th2 response via ST2 blockade	Mouse	63
Ova-induced food allergy	*B. longum* KACC 91,563 with IgE_TRAP_	(+) neutralizes IgE and ↓ allergic responses	Mouse	64
Ova-induced rhinitis	*B. breve*	(+) ↑ levels of splenic Treg along with ↓ Th2 responses (serum IgE, IL-4, and IL-10)	Mouse	65

DSS = Dextran Sodium Sulfate; EAE = experimental autoimmune encephalomyelitis; EAMG = experimental autoimmune myasthenia gravis

### DSS colitis

Various species and strains of *Bifidobacterium* are linked to the downmodulation of dextran sodium sulfate (DSS)-induced colitis in mice, including *B. pseudocatenulatum* MY40C and CCFM680,^45^ *B. infantis* CGMCC0460.1,^46^ *B. animalis* subsp. *lactis* XLTG11,^47^ and *B. breve* M1, M2, M3, and M4.^48^ DSS is a water-soluble, negatively charged polysaccharide that causes intestinal damage and inflammation, closely resembling UC in humans. Colitic mice treated with *B. infantis* CGMCC0460.1 or *Bifidobacterium animalis* subsp. *lactis* XLTG11 show a reduction in disease as assessed by weight loss, colon length, histologic tissue damage, myeloperoxidase activity, gut permeability, spleen weight, and disease activity index.^46,47^ Mechanistically, *B. pseudocatenulatum* MY40C and CCFM680, *B. infantis* CGMCC0460.1, *B. animalis* subsp. *lactis* XLTG11, and *B. breve* M1 and M2 but not M3 and M4 treatment results in upregulation of tight junction and adherens junction proteins (β-catenin, claudin-3, occludin, and ZO-1) along with increased barrier modifying mucin 2, IL-10, and PPARγ, while downregulating pro-inflammatory TNFα and IL-6.^45–48^ *B. pseudocatenulatum* MY40C and CCFM680 and *B. animalis* subsp. *lactis* XLTG11 also downregulate the TLR4/NFκB pathway in colitic animals, reducing immune responsiveness to gut microbes and shifting the host immune state from inflammation to homeostasis.^45,47^ Although the mechanism underlying these effects remains poorly understood, production of colonic conjugated linoleic acid, an anti-inflammatory polyunsaturated fatty acid, also increased in mice treated with *B. pseudocatenulatum* MY40C and CCFM680 or *B. breve* M1, M2, M3, and M4.^45,48^ Additionally, treatment of DSS-induced colitic mice with a mixture of *Bifidobacterium* species (*B. bifidum, B. longum, B. lactis*, and *B. breve*) results in an altered host gut microbiome in a Treg dependent fashion.^49^ *Bifidobacterium* treatment also resulted in enhanced Treg function via promotion of a self-stimulatory IL-10/IL-10 Rα loop and upregulation of mitochondrial activity. Of the *Bifidobacterium* mixture, *B. breve* specifically ameliorates CTLA-4 blockade-induced colitis, also via enhanced Treg function (Table 2).

### Gliadin-induced enteropathy

Bifidobacteria protect intestinal epithelial cells from damage in a gliadin-induced enteropathy (GIE) model of celiac disease, in which incomplete hydrolysis of dietary proteins leads to small intestinal inflammation, lymphocyte infiltration, villous atrophy, and crypt hyperplasia.^50^ Specifically, treatment of mice with *B. longum* CECT 7347 partially suppresses disease by inhibiting the production of inflammatory cytokines and CD4 T cell mediated immune responses. Similar anti-inflammatory effects are observed with *B. longum* ES1 and *B. bifidum* ES2 *in vitro*.^53^ While *B. longum* CECT 7347 restores intestinal structure without reversing cellular infiltration,^50^ *B. longum* NCC2705 treatment prevents intraepithelial infiltration of lymphocytes in mice sensitized with gliadin.^52^ Further, in culture with epithelial cells, *B. lactis* counteracts the gliadin-induced permeability of intestinal epithelium, inhibited membrane ruffle formation, and protected tight junctions.^54^ While GIE leads to decreased NFκB and increased TNFα and IL-10 expression, *B. longum* CECT 7347 treatment restores baseline NFκB and IL-10 levels, but further increases TNFα.^50^ *B. longum* CECT 7347 treatment also modulates T cell differentiation by reducing overall CD4 and Treg populations while increasing CD8 T cells.^50^ In another study, *B. longum* CECT 7347 co-administration with gliadin to IFNγ-sensitized mice results in upregulated stress and intestinal absorption proteins along with downregulated cellular homeostasis proteins involved in cytoskeletal organization, protein transport, gene transcription, retinoic acid binding, and cell starvation.^51^ These observations reinforce the notion that the effects of *Bifidobacterium* treatment are pleiotropic and context dependent (Table 2).

### Experimental autoimmune myasthenia gravis

Administration of *Bifidobacterium*, alone or in combination with other probiotics, ameliorates symptoms (clinical score, weight loss, and body trembling) of experimental autoimmune myasthenia gravis (EAMG) in rats.^55,56,66^ Serum nicotinic acetylcholine receptor autoantibodies, elevated in EAMG models, are decreased in *Bifidobacterium-*treated rats.^55,56,66^ Pro-inflammatory IFNγ, TNFα, IL-6, and IL-17 are downregulated in the thymus of *Bifidobacterium*-treated EAMG rats.^55^ Furthermore, naïve rats treated with *B. animalis subsp. Lactis* BB12 and LMG S-28195 have increased Tregs in Peyer’s patches, mesenteric LNs (MLN), and in peripheral blood leukocytes. *B bifidum*, as part of a probiotic cocktail consisting of *Lactobacillus casei*, *Lactobacillus acidophilus, Lactobacillus reuteni*, and *Streptococcus thermophilus*, also skews *in vitro* DCs toward a regulatory phenotype with increased expression of IL-10, TGFβ, arginase 1, and aldh1a2.^56^ Treatment with a mixture of *B. animalis* subsp. *lactis* BB12 and *B. animalis* subsp. *lactis* LMG S-28195 modifies the diversity of EAMG animal microbiomes, reducing disease-associated dysbiosis.^66^ While largely associative, these studies point to *Bifidobacterium* as a critical mediator of neuroimmune homeostasis (Table 2).

### Experimental autoimmune encephalomyelitis

Similar to EAMG, *Bifidobacterium* also ameliorates experimental autoimmune encephalomyelitis (EAE) in rats.^55^ Myelin basic protein (MBP)-immunized rats, after injection with MBP-specific T cell blasts, have fewer of these cells localized to spinal cord tissue after treatment with *B. animalis subsp. Lactis* BB12 and LMG S-28195. *Bifidobacterium* in combination with *Lactobacillus* or as part of a larger cocktail (*B. bifidum*, *Lactobacillus casei*, *Lactobacillus acidophilus*, *Lactobacillus reuteni*, and *Streptococcus thermophilus*) prevents the progression of EAE in mice.^57,58^ EAE and multiple sclerosis share many clinical and pathological features, with pathogenesis dependent on IL-17-producing T cells.^58^ Probiotic-treated EAE mice show reduced central nervous system inflammation with limited neuronal demyelination. Treatment also increases Tregs in peripheral LN (PLN) and spleen while inhibiting Th1 and Th17 polarization. IL-17, IL-6, and IFNγ are downregulated, while TGFβ, IL-4, and IL-10 are upregulated.^57,58^ In contrast, *Bifidobacterium* is enriched in patients with multiple sclerosis in both pediatric and adult human case–control series^59,60^ (Table 2).

### Food allergies

Bifidobacteria are more commonly found in the gut microbiomes of individuals who do not suffer from food allergies, suggesting the importance of Bifidobacteria in regulating allergic responses. In a mouse model of shrimp tropomyosin-induced allergy, treatment with either *B. lactis* or *B. infantis* 14.518 reduces allergic symptoms (including decreased serum IgE in both children with food allergies and mouse allergy models).^61,62^ Mice treated with *B. lactis* exhibit increased Treg/Th17 ratios^61^. Increased microbiome *Dorea* and decreased *Ralstonia* in treated animals correlates with elevated Treg/Th17 ratios, suggesting their involvement in the immunomodulatory response induced by *Bifidobacterium* administration. *B. infantis* 14.518 increases DC maturation and CD103+ tolerogenic DC accumulation in Peyer’s patches and MLN. This leads to a similar increase in Tregs and suppression of Th2 responses.^62^ *B. infantis* 14.518 partially restores gut microbiome richness in tropomyosin-sensitized animals.^62^ In mice with ovalbumin (Ova)-induced food allergy, gut dysbiosis, inflammation, and inflammatory cell infiltration are reduced or inhibited by concurrent treatment with *B. breve M*-16 V.^63^ The probiotic modulates immune responses by inhibiting Th2 responses via ST2 blockade.^63^ A similar study on the effects of *B. breve* administration on Ova-induced rhinitis shows increased levels of splenic Treg and decreased Th2 responses, including serum IgE, IL-4, and IL-10.^65^ *B. longum* KACC 91,563 also synergizes with IgETRAP, a fusion protein of human high-affinity IgE receptor extracellular domain, hFcεRI, and an IgD/IgG4 hybrid Fc domain, to neutralize IgE and alleviate allergic responses in an Ova-induced food allergy model, including hypothermia, anaphylaxis score, serum IgE and MCPT-1, mast cell numbers, and goblet cell hyperplasia.^64^ This combined therapy does not alter the host microbiome, suggesting that *B. longum* KACC 91,563 does this without host colonization^64^ (Table 2).

## PART IV. *Bifidobacterium* cell surface components serve as immunomodulators

### Exopolysaccharides

**Figure 1.**
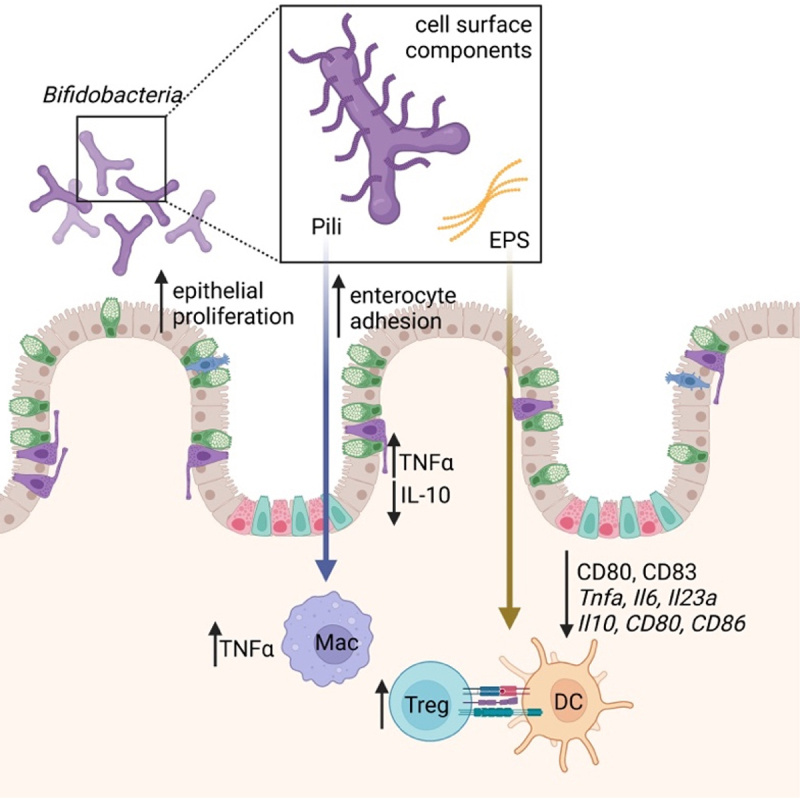
*Bifidobacterial* cell surface components, present in both attached and secreted forms, within gut lumen. These components act on both gut epithelium (increasing proliferation, enterocyte adhesion, and modulating cytokine production) and host immune cells (DC, macrophages, and Tregs). DC = dendritic cell; EPS = exopolysaccharide; Mac = macrophage; Treg = Foxp3+ regulatory T cells.

Exopolysaccharides (EPS) are carbohydrate polymers expressed on the cell surface or secreted by bacteria for both protection and interaction with the surrounding environment.^67,68^ Reflected by the large inter- and intra-species variability in gene clusters responsible for EPS biosynthesis, structure, and composition, these molecules play numerous roles in host–microbe interactions, including adhesion to the intestinal epithelium and protection from adverse environmental conditions.^69,70^ In *Bifidobacterium*, EPS are also implicated in the modulation of host immune responses (Figure 1).^67,70–72^ Knocking out EPS expression in *Bifidobacterium breve* enhances the DC inflammatory phenotype by increasing the expression of co-stimulatory molecule genes *Cd80* and *Cd83*.^18^ Cell surface β-glucan/galactan polysaccharides of *B. bifidum* PRI1 induce immunosuppressive Tregs via Toll-like receptor (TLR) 2 signaling on regulatory DCs.^6^ Even between strains of the same *Bifidobacterium* species, EPS structure and immune regulation differ. For example, *B. breve* UCC2003 has a thicker EPS layer and more anti-inflammatory phenotype than *B. breve* JCM7017 through modulation of macrophage IL-10 and TNFα and DC *Tnfa, Il6*, and *Il23a* expression.^18^ Murine *B. pseudolongum* UMB287 MBP-01 EPS increases intestinal Tregs compared to control, but EPS from porcine-derived *B. pseudolongum* ATCC25526 did not. EPS from both strains results in increased intestinal DC, MLN DC, and MLN macrophages.^73^ The cell surface components of *B. longum* strains NCC 2705, ATCC 15,707, and BIF53, but not BB536 or NCIMB 8809, stimulate the production of IL-10 and TNFα in isolated peripheral blood mononuclear cells.^74^ Given the context-dependence of bacterial EPS expression, its effects are likely only a portion of the *Bifidobacterial* immune modulatory mechanism (Table 3).

**Table 3.** *Bifidobacterium* cell surface components and their immunomodulatory properties.

Cell Surface Component	Strain/Genus	Presence (+) or Absence (-) of Cell Surface Component Biomolecular/Cellular Effects	References
Cell surface β-glucan/galactan polysaccharides	*B. bifidum* PRI1 β-glucan/galactan polysaccharides	(+) ↑ immunosuppressive Tregs via Toll-like receptor 2 signaling on regulatory DCs	^6^
EPS	*B. breve* UCC2003 and JCM7017	(-) ↑ DC maturation and activation ↑ anti-inflammatory phenotype ↑ *Cd80, Cd83, Cd86* expression	18
EPS	*B. breve* UCC2003	(-) ↑ MΦ IL-10, TNFα ↑ DC *Tnfa, Il6, Il12a, Il23a*	18
EPS	*B. breve* JCM7017	(-) ↓ MΦ IL-10, TNFα	18
EPS	*B. pseudolongum* UMB287 MBP-01	(+) ↑ intestinal Tregs, DC ↑ MLN DC, MΦ	73
EPS	*B. pseudolongum* ATCC25526	(+) ↑ intestinal DC ↑ MLN DC, MΦ	73
Cell surface components	*B. longum* NCC 2705, ATCC 15,707, and BIF53	(+) ↑ IL-10, TNFα, IFNγ in isolated PBMCs	74
SD pili	*B. bifidum* PRL2010 SD pili expressed on non-piliated *Lactococcus lactis* NZ9000	(+) ↑ adhesion to human intestinal enterocytes ↑ TNFα ↓ IL-10	69,75
Tad pili	*B. breve* UCC2003 Tad pili	(+) ↑ colonic epithelial proliferation ↑ crypt Ki67+ cells	76

EPS = exopolysaccharide; DS = sortase-dependent pili; Tad = type IVb tight adherence pili

### Pili

Pili are surface appendages that mediate adhesion to the host intestinal epithelium, bacterial cell aggregation, motility, electron transfer, biofilm formation, and immunomodulation.^77^ These structures display bacterial species-specific immunomodulatory properties.^69^ *Bifidobacteria* utilize sortase-dependent (SD) pili, consisting of covalently cross-linked pilin monomers anchored to the cell wall.^69,76^ These SD pili have roles in virulence, nutrient acquisition, mucin production, host adhesion, and immune signaling (Figure 1).^78^ *Bifidobacteria* also express type IVb tight adherence (Tad) pili, which mediate host surface adhesion.^76^ Expression of the *B. bifidum* PRL2010 SD *pil*3_PRL2010_ gene cluster in the normally non-piliated *Lactococcus lactis* NZ9000 enhances adhesion to human intestinal enterocytes and evokes increased TNFα in a human macrophage-like cell line and mouse model compared to non-piliated control bacteria.^75^ Oral administration of piliated *L. lactis*-pil3PRL2010 results in decreased IL-10 compared with non-piliated controls in mice.^75^ Although *B. bifidum* PRL2010 pili induce higher TNFα expression, they act as weak inducers of other systemic pro-inflammatory cytokines such as IL-12,^69,75^ suggesting that the immunomodulatory effects of SD pili may be limited to local mucosal immune responses.^75^ Given the presence of *B. bifidum* PRL2010 in the infant gut microbiome, the local pro-inflammatory effects of *B. bifidum* PRL2010 SD pili may prime the neonatal immune system^69,75^ (Table 3).

## PART V. Metabolites directly produced by *Bifidobacterium* act as immune mediators

### Acetate

Metabolites from *Bifidobacterium* carbohydrate fermentation directly and indirectly influence host immune responses.^69^ Acetate is one of the most abundant metabolites produced by *Bifidobacterium longum* 5^1A^ reaching the systemic circulation.^79^ Acetate modulates host defenses and provide protection against various diseases. Mice pre-treated with live *B. longum* 5^1A^ or with acetate have elevated levels of IL-10 in lung tissue following *Klebsiella pneumoniae* infection compared to untreated infected mice, thus protecting the lungs from injury.^79^ The ability of *Bifidobacterium* species and strains to produce acetate varies considerably.^80^ This variability is influenced not only by the specific species or strain of *Bifidobacterium*, but also by the nutrient environment in which these bacteria reside. The specific genetic composition of each *Bifidobacterium* strain confers it with the capability to metabolize a different range of carbohydrate types, directly shaping its metabolic potential. For example, colonization of germ-free mice with *B. longum* subsp. *longum* JCM 1217 or *B. longum* subsp. *infantis* 157F results in significantly higher concentrations of fecal acetate than colonization with *B. longum* subsp. *infantis* JCM 1222^T^ or *B. adolescentis* JCM 1275^T81^. Colonization with the former two strains conferred increased survival in mice inoculated with Shiga toxin-producing enteropathogenic *E. coli* O157.^81^ These *B. longum* strains increase acetate production via expression of ATP-binding cassette (ABC)-type carbohydrate transporters that increase sugar consumption for catabolism and acetate production, explaining how these bacteria can still produce acetate even in the fructose-limited distal colon.^81^ Protection against lethal enteropathogenic *E. coli* O157:H7 infection in the colon by *Bifidobacterium longum* subsp. *longum* JCM 1217^T^ is dependent on acetate production in the distal colon. This is accompanied by upregulation of host immune modulating genes (Apoe, C3, and Pla2g2a) and prevention of Shiga toxin translocation from the gut to the circulation.^81,82^ This also demonstrates how different strains of *B. longum* subsp. *infantis*, in addition to different *Bifidobacterium* species, can induce differential gene expression related to acetate production, leading to a spectrum of downstream immune effects (Table 4).

**Table 4.** *Bifidobacteria-*produced metabolites and immunomodulatory properties.

Metabolite	Regulatory Mechanism	Strain	Model/System	References
Acetate	↑ protective IL-10 in lung	*B. longum* 5^1A^	*Klebsiella pneumoniae* infection	79
Acetate	↑ survival of infected mice; ↑ host immune modulating genes (Apoe, C3, and Pla2g2a); ↓Shiga toxin translocation	*B. longum* subsp. *longum* JCM 1217T	*E. coli* O157:H7 infection	81,82
Lactate	↓ DC/macrophage inflammatory responses to TLR4 and TLR9 engagement (via GPR81); ↓ hepatitis and pancreatitis	*B. animalis* ATCC25527	LPS/galactosamine-induced hepatitis; LPS-induced pancreatitis; intraperitoneal caerulein/retrograde pancreatic duct sodium taurocholate pancreatitis	83,84
CLA	protects gut microbiota from redox damage	*B. breve* CCFM683, UCC2003, DSM 20,213	In vitro bacteria culture	85,86
IsoalloLCA (secondary BA)	↑ mitochondrial reactive oxygen species, FOXP3 expression, Treg differentiation	*B. pseudocatenulatum*	*In vitro* primary mouse cells and human cell lines; screen of human stool isolates	87,88
ILA (Tryptophan metabolite)	↓ IL-8 in IL-1β stimulated human H4 immature primary small intestinal epithelial cell lines; ↓ LPS-induced IL-8 in Caco-2 intestinal epithelial cell line; ↓ LPS-induced NFκB activation in murine RAW-blue macrophage reporter cell line, engineered with secreted embryonic alkaline phosphatase	*B. longum* subsp. *infantis* ATCC 15,697	*In vitro* treated human and mouse cell lines	89,90
I3C (Tryptophan metabolite)	↓ atopic dermatitis symptoms in mice/humans via ↓ Th2 immune responses through AhR activation	*B. longum* CCFM1029	Mouse model of atopic dermatits; analysis of AD patient serum/stool	91
IAA (Tryptophan metabolite)	↑ Foxp3 and ↓ RORγt/STAT3 via AhR signaling	*Bifidobacteria*	Proteoglycan-induced ankylosing spondylitis mouse	92
Inosine	↑ anti-tumor immunity via modulation of anti-CTLA4 (immune checkpoint blockade); mediates CD4 Th1 differentiation via IFN-γ signaling through T cell A_2A_R; ↑ CD4 T cell IL-12R, engaged by conventional DC-produced IL-12	*B. pseudolongum*	MC38 tumor cell injection into mice; Mice bearing B16-melanoma; immune-deficient mice bearing GD2-positive human neuroblastoma (LAN-1) xenografts	93,94

CLA = Conjugated Linoleic Acid; I3C = indole-3-carbaldehyde; IAA = indole-3-acetic acid; ILA = indole-3-lactic acid; isoalloLCA = isoallolithocholic acid; TLR4/9 = Toll Like Receptors 4 & 9

### Lactate

Unlike acetate, lactate production is independent of the nutrient environment and relies only on the presence of bacterial strains.^80^ *B*. *kashiwanohense* DSM21854, *B*. *gallicum* DSM 20,093, and *B*. *longum infantis* 157 F NC produce acetate and lactate. Polysaccharides including starch, inulin, and arabinoxylab via the DC and macrophage receptor GPR81, blunting the inflammatory responses of cells to TLR4 and TLR9.^83^ Treatment with lactate results in decreased LPS- and galactosamine-induced hepatitis and LPS-induced pancreatitis. Furthermore, in a murine pancreatitis model using intraperitoneal caerulein or retrograde sodium taurocholate injection into the pancreatic duct, *B. animalis* ATCC25527 and its metabolite lactate, exert a protective effect.^84^ This is demonstrated through serum amylase reduction, reduction of pancreatic lesions, and improved survival (Table 4).

### Conjugated linoleic acid

Bifidobacterium-mediated conversion of linoleic acid (LA) into conjugated linoleic acid (CLA) is critical for the maintenance of intestinal homeostasis.^85^ LA induces redox stress and reduces growth of many different bacterial species, resulting in widespread metabolic reprogramming and dysbiosis.^86^ However, several Bifidobacterial strains are capable of converting free LA into CLA isomers.^85,86^ While the exact mechanism behind this bioconversion is unknown, production of CLA promotes gut homeostasis by protecting the microbiome from LA accumulation, which has been shown to confer protective effects in a number of disease models as well as to promote an anti-inflammatory environment^85,86,95^ (Table 4).

### Inosine

The purine nucleoside inosine has best been characterized for its role as an anti-tumor immunomodulator.^93^ Inosine is produced by *B. pseudolongum* and enhances anti-tumor immunity via modulation of immune checkpoint blockade by anti-CTLA4 treatment. Inosine mediates CD4 Th1 differentiation via IFN-γ signaling through A_2A_R on T cells. Immune checkpoint blockage (ICB) therapy is associated with elevated intestinal barrier permeability, which potentially facilitates translocation of inosine and other metabolites from the gut lumen into the systemic circulation. This augmented systemic translocation of metabolites may underpin the observed systemic effects of *B. pseudolongum* in the context of ICB therapy. Inosine also upregulates the IL-12 receptor on CD4 T cells, which is engaged by conventional DC-produced IL-12^89^ (Table 4).

### Tryptophan

Tryptophan metabolism is an important mediator of *Bifidobacterial* immune modulation. *Bifidobacterium longum* subsp. *infantis* ATCC 15,697, a “star colonizer” in breastmilk fed infant gut, produces aromatic lactic acids such as indole-3-lactic acid (ILA) from metabolizing tryptophan, which reduces inflammatory IL-8 production in the human H4 immature primary small intestinal epithelial cell line after IL-1β stimulation.^89,96^ Similarly, ILA pre-treatment significantly decreases LPS-induced IL-8 production in the Caco-2 intestinal epithelial cell line.^90^ ILA production is enriched in *B. infantis* ATCC 15,697 grown on HMO-supplemented media compared with lactose-supplemented media, suggesting that ILA production is driven by the metabolism of milk glycans.^90^ ILA pre-treatment inhibits LPS-induced NFκB activation in a dose-dependent manner in the murine RAW-blue macrophage reporter cell line, engineered with secreted embryonic alkaline phosphatase.^90^ ILA signals through the aryl hydrocarbon receptor (AhR), which plays a role in regulating intestinal homeostasis and crosstalk with the cytoprotective Nrf2 pathway that reduces oxidative stress^90^ (Table 4).

**Figure 2.**
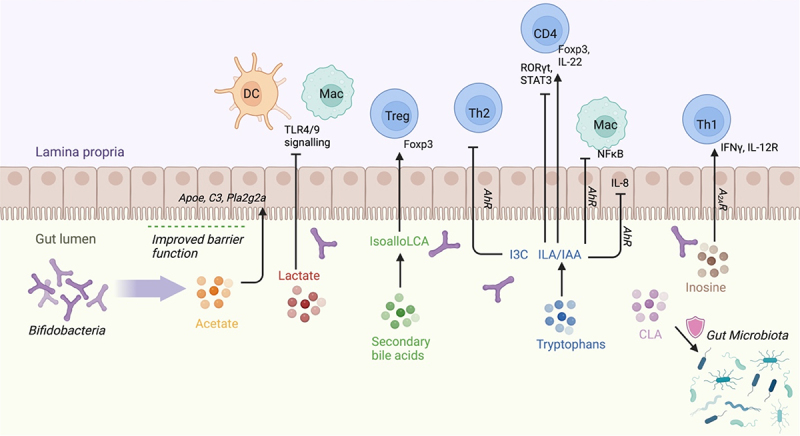
*Bifidobacterium-*derived metabolites modulate transcription factors and cytokine production in immune cells as well as in gut epithelium.

Aromatic amino acid-derived aromatic lactic acids are ligands for AhR that influence gut homeostasis through enhanced mucosal barrier function, protection from pathogens,^97^ and host metabolism.^98^ *Bifidobacterial* species convert aromatic amino acids (tryptophan, phenylalanine, and tyrosine) into their respective aromatic lactic acids (ILA, phenyllactic acid, and 4-hydroxyphenyllactic acid), which in turn activates AhR.^96^ Many *Bifidobacterium*-derived aromatic lactic acids are found in the infant gut from *B. longum, B. bifidum*, and *B. breve*.^96^ The ability of each species to produce aromatic lactic acids is tied to its ability to use HMOs as a carbohydrate source. In particular, ILA is enriched in the gut of breastfed infants, which modulates IL-22 production in CD4 T cells *in vitro* via AhR. *B. longum* CCFM1029 administration increases levels of the tryptophan metabolite indole-3-carbaldehyde (I3C) and improves atopic dermatitis symptoms in mice and humans by suppression of Th2 type immune responses via AhR activation.^91^ Indole-3-acetic acid (IAA) is another tryptophan metabolite and AhR ligand produced by *Bifidobacterial* species.^99^ In ankylosing spondylitis mice, IAA restores the ileal lamina propria Th17/Treg balance via AhR, resulting in increased levels of Foxp3 and downregulation of RORγt and STAT3 (Figure 2).^92^ However, there is a broad array of microbiota-derived molecules that act as AhR modulators apart from aromatic lactic acids. For example, the production of butyrate, which can be stimulated by *Bifidobacterium* as mentioned above,^19^ acts as an HDAC inhibitor in human intestinal epithelial cell lines and colonic biopsies, increasing the recruitment of AhR to the target gene promoter in the presence of tryptophan-derived AhR agonists,^100^ Furthermore, tryptophan metabolite indole-3-aldehyde (I3A), catabolized by *Lactobacillus reuteri*, found within the melanoma tumor microenvironment, facilitates immune checkpoint inhibition via AhR.^101^

AhR = aryl hydrocarbon receptor; CD4 = CD4 Helper T cell; CLA = Conjugated Linoleic Acid; DC = dendritic cell; I3C = indole-3-carbaldehyde; IAA = indole-3-acetic acid; ILA = indole-3-lactic acid; isoalloLCA = isoallolithocholic acid; Mac = macrophage; SCFA = short-chain fatty acids; TLR4/9 = Toll Like Receptors 4 & 9; Treg = Foxp3+ regulatory T cells

### Vitamins

*Bifidobacterium*, along with lactic acid fermenting bacteria, have been reported to synthesize B-group and K-group vitamins de novo, providing an estimated 30% of the host’s daily intake.^102^ Notably, several *Bifidobacterium* species are known to produce B vitamins, including B2 (riboflavin), B6, B9 (folate), and B12 (cobalamin). These vitamins are vital cofactors in numerous metabolic processes, including those integral to host immune function.^103^ The impact of these vitamins on the immune system is multifaceted: they aid in the development and function of lymphocytes, modulate cytokine production, and support the integrity of the mucosal barriers, forming the first line of defense against pathogens. Furthermore, *Bifidobacterium*‘s role in vitamin synthesis extends beyond B and K vitamins. A recent study showed commonly used human probiotic strain *Bifidobacterium bifidum*, along with other bacteria from *Bacilli* and *Clostridia*, converts dietary vitamin A to retinoic acid (RA) via aldehyde dehydrogenases.^104,105^ The upregulated RA-dependent responses in intestinal epithelial cells can provide protection against pathogen colonization, and crucially, contribute to both immunological tolerance and the elicitation of adaptive immune responses.^106^ For example, RA generation from vitamin A occurs in intestinal epithelial cells and a subset of DCs, resulting in the conversion of naïve T cells into Tregs.^107^ The vitamin-producing capability of *Bifidobacterium* thus not only underscores its importance in maintaining gut health, but also highlights its broader influence in immune regulation.

## *PART VI. Bifidobacterium*-influenced metabolites as immunomodulators

While *Bifidobacterium* does not possess all the metabolic machinery to produce all known immunomodulatory metabolites, it does contribute to an environment permissive toward production of these metabolites by other pro-homeostatic bacteria through mechanisms such as cross-feeding (Table 5).

**Table 5.** *Bifidobacteria*-influenced metabolites and immunomodulatory properties.

Metabolite	Regulatory Mechanism	Influence	Model/System	References
Butyrate	↑ TGFβ and IL-10 expression (APCs/IECs); ↑ Treg development; colorectal cancer cell apoptosis	*Bifidobacterium*-produces acetate, which drives *Anaerostipes caccae* and *Eubacterium rectale* ATCC 33,656 production of butyrate via butyryl-CoA:acetate CoA-transferase	*In vitro* and *in vivo* mouse Treg differentiation; tumor cell line	19,20,108,109
isoDCA (secondary BA)	↓ TNFα and IL-6 in DCs; ↑ Foxp3 induction, peripheral Tregs	*Bifidobacterium* cannot directly produce isoDCA, but encodes BSH that deconjugates BAs into other secondary BAs which may have similar immunomodulatory roles	*In vitro* mouse T cell/DC culture; *in vivo* mouse metabolite treatment	110–112
Spermine	↓ M1 macrophage activation via suppression of ornithine decarboxylase and ↓ pro-inflammatory cytokine synthesis	*Bifidobacterium* enhances polyamine production *from E coli* and *E. faecalis* via luminal acidification	Human PBMCS, mouse macrophage cell lines	33,113,114

### Butyrate

Butyrate regulates the expression of anti-inflammatory cytokine genes, such as TGFβ and IL-10, in antigen presenting cells (APCs) and intestinal epithelial cells (IECs) and stimulates Treg development.^108,109^ Butyrate has anti-oncogenic properties through induction of tumor cell apoptosis in colorectal cancer.^108^ Similarly, acetate induces genes involved in anti-inflammatory responses^81^ and acts on the colonic epithelium to enhance barrier function, thereby blocking the translocation of pathogens and toxins (Figure 2).^81^ While *Bifidobacteria* are unable to directly synthesize butyrate, *Bifidobacterium* and the acetate they produce may influence the activity and composition of other members of the gut microbiota that produce butyrate, thus stimulating a secondary butyrogenic effect.^19,69,115^ Co-culture of *B. longum* subsp. *longum* NCC2705, an arabinoxylan oligosaccharide (AXOS)-converting acetate producer, and *Eubacterium rectale* ATCC 33,656, an acetate-converting butyrate producer, in a growth medium with AXOS yields *Bifidobacterium* proliferation and butyrate production.^19^ This occurs due to butyryl-CoA:acetate CoA-transferase upregulation in *E. rectale* ATCC 33,656, which utilizes acetate as a co-substrate in the final step of butyrate biosynthesis (Table 5).

### Secondary bile acids (BAs)

Secondary BAs also contribute to the regulation of immune homeostasis that are mostly known to be indirectly influenced by *Bifidobacterium* via modulating the gut environment and the microbial community structure^116^. Certain *Bifidobacterium* species can deconjugate bile salts, which can then influence the ability of other bacteria such as *Bacteroides, Eubacterium, Ruminococcus, Clostridium*, and *Escherichia* to further metabolize these bile components into secondary bile acids. The conversion of primary to secondary bile acids is a key process in the gut, which plays a significant role in maintaining gut homeostasis in addition to influencing metabolic and immune functions. The interplay between *Bifidobacterium* and these other bacterial species in the metabolism of bile components highlights the intricate and collaborative nature of the gut microbiome. Derived from cholesterol catabolism in the liver, primary BAs are secreted postprandially into the duodenum to aid the uptake of dietary fatty acids and fat-soluble vitamins.^87,117^ Most BAs return to the liver from the gut via the enterohepatic circulation. However, approximately 5% of BAs escape reabsorption in the ileum and are modified by resident enteric bacteria into secondary BAs via dehydroxylation, dehydrogenation, and deconjugation.^87,110,117^ Secondary BAs can signal Tregs, Th17, or DCs in immune-mediated disorders.^87,118,119^

*Bifidobacterium* have been identified in a high-throughput screen of human stool as a converter of 3-oxolithocholic acid (3-oxoLCA), a BA found in the human gut, to isoallolithocholic acid (isoalloLCA), a known immunomodulatory secondary BA.^87^ IsoalloLCA increases the rate of cellular oxygen consumption, inducing mitochondrial reactive oxygen species that increases the expression of FOXP3, thereby increasing Treg differentiation.^88^ Despite these connections, few studies have directly investigated the production of secondary BAs by *Bifidobacterial* species or determined their effects on immune regulation (Table 5).

Anti-inflammatory secondary BA 3β-hydroxydeoxycholic acid (isoDCA) decreases DC TNFα and IL-6 production.^110^ IsoDCA also promotes Foxp3 induction, increasing the number of peripheral Tregs (Figure 2). Although *Bifidobacterium* lack 7α-dehydroxylation activity to produce isoDCA from cholic acid.^120^ *Bifidobacterium* encode bile salt hydrolases that deconjugate BAs into other secondary BAs that may have similar immunomodulatory roles.^111,112^

### Polyamines

Polyamines (i.e., putrescine, spermidine, and spermine) play a critical role in regulating immunity and inflammation.^121,122^ While *Bifidobacterium* does not possess polyamine biosynthetic machinery, its presence does enhance the production of polyamines from other bacterial sources. Administration of several different strains of *Bifidobacterium* can increase intralumenal putrescene via acidification, likely through acetate and lactate production, which augments putrescine production by *E. coli* and *Enterococcus faecalis* ^33^. Spermine restrains innate immune responses by inhibiting M1 macrophage activation via suppression of ornithine decarboxylase and pro-inflammatory cytokine synthesis without perturbing anti-inflammatory TGFβ and IL-10 (Figure 2).^113,114^ Spermidine also modulates systemic and mucosal adaptive immunity by modulating T cell differentiation.^122,123^ Clinically, N-acetyl putrescine and N-acetyl spermidine are enriched in allo-hematopoietic stem cell transplantation (HSCT) recipients free from graft versus host disease (GvHD) compared to those with GvHD^124^ (Table 5).

## PART VII. Future directions: therapeutics and technological pipeline

Initial investigations into the role of *Bifidobacterium* in human disease has been predominantly associative, yet recent studies have begun to delve into the immunomodulatory mechanisms and causal role *Bifidobacterium* plays in host immune responses. Recognizing a causal relationship not only lays the groundwork for the formulation of targeted therapeutic strategies but also illuminates the underpinnings of disease pathogenesis. This enhanced understanding paves the way for novel drug development, refined disease progression predictions, and innovative preventative strategies.

Though *Bifidobacterium* displays immunologic pleiotropy, varying across host species and specific strains, there is an increasing interest in its surface components and derived metabolites. These components present promising targets for host immune modulation. A better understanding of the metabolic perturbations due to *Bifidobacterium* and the influence of their metabolic products on pro-tolerant and pro-homeostatic immunity are critical, especially when considering clinical scenarios such as solid organ transplantation, autoimmunity, and anti-tumor responses. Furthermore, a deeper understanding of the role *Bifidobacteria* within the broader commensal gut microbiota communities is pivotal. Such knowledge will be instrumental in optimizing live biotherapeutics for the prophylaxis or treatment of disease conditions.

### Live biotherapeutics

The absence of HMO-metabolizing *Bifidobacterium* in the infant gut correlates with Th2- and Th17-driven inflammation in the intestine, as well as both acute and chronic systemic immune disorders.^8,125^ Investigators have begun to test the impact of exogenous Bifidobacteria on improving overall immune health. Infants fed a diet supplemented with *B. longum* subsp. *infantis* EVC001, an optimized strain containing all HMO-utilization genes, display a higher abundance of *Bifidobacterium* within two months of administration.^125,126^ These infants have decreased intestinal inflammation, as evidenced by decreased fecal inflammatory cytokine and calprotectin levels^125^. Fecal water from EVC001-treated infants also skews the polarization of naïve T cells cultured under Th0 conditions toward a Th1-like state, whereas fecal water from infants lacking *B. infantis* induces a Th2-like state. When cultured under Th17 polarizing conditions and exposed to EVC001 fecal water, T cells show reduced levels of activation and proliferation markers compared to controls. This effect is recapitulated by *B. infantis*-derived metabolite ILA, which in addition to the effects mentioned above, upregulates CXCR3, granzyme B, and galectin-1 in cultured cells.^8^ Administration of EVC001 is also associated with an increase in intestinal IFNβ, a known inducer of Tregs.^8,127^ Along with changes in cytokine and immune responses, EVC001 supplementation contributes to reducing the amount of virulence factors in the infant gut, restricting the establishment of pathogenic bacterial communities.^126^ This reinforces the importance of *Bifidobacterium*-derived metabolites as a mechanism of immunomodulation and as potential therapeutic avenues.

In adults with active UC, one month of treatment with *B. longum* and prebiotic (preferential inulin-oligofructose growth substrate) reduces endoscopic and histologic inflammation in the colon and reduces mucosal TNFα and IL1 mRNA levels.^128^ *Ex vivo*, heat-killed *B. breve* strain Yakult and *B. bifidum* strain Yakult both induce increased IL-10 levels in peripheral blood mononuclear cells from UC patients.^129^ Similarly, in CD patients, *B. longum* and prebiotic reduces disease activity indices, histologic scores, and mucosal TNFα levels.^130^ *B. infantis* 35624 demonstrates a clinical anti-inflammatory effect in UC, chronic fatigue syndrome, and psoriasis. After 6–8 weeks of administration, this probiotic strain reduces plasma CRP levels in all three conditions, reduces TNFα in chronic fatigue syndrome and psoriasis, and reduces IL-6 in UC and chronic fatigue syndrome.^131^

Although there has been much interest and studies of the benefits of *Bifidobacteria*-based probiotics, colonization of strains and the direct mechanisms underlying these effects have been challenging to elucidate. Given the complex interactions that *Bifidobacteria* has with other members of the gut microbiome, off target effects of ongoing probiotic treatment are likely. However, small molecule mediators of the immune modulatory effects of *Bifidobacterial* strains have a demonstrated potential as therapeutics that have higher purity with more direct and consistent effects than probiotics. Furthermore, metabolites may be able to be administered in a more targeted, tissue-specific manner, compared to probiotics, which must be administered enterally. Future studies will necessarily need to focus on the tissue-specific effects of probiotics, but more specifically of individual metabolic alterations in response to probiotics.

### Anti-tumor immunotherapy enhancement

In addition to its direct role in the treatment of autoimmune and inflammatory diseases, *Bifidobacterium* has also been implicated in modulating the effects of anti-cancer regimens and enhancing anti-tumor immunity. From a mechanistic standpoint, some of these effects may be mediated by *Bifidobacterium*-derived metabolites. *B. pseudolongum* promotes immune checkpoint blockade efficacy in mouse models of melanoma, bladder cancer, and colorectal cancer through its production of inosine, which acts on T cells via A_2A_R.^94^ In humans, stool samples from patients with improved clinical responses to anti-programmed cell death protein 1 (αPD-1)-based immunotherapy for metastatic melanoma show *Bifidobacterium longum* enrichment.^132^ Administration of a *Bifidobacterial* cocktail containing *B. breve* and *B. longum*, both alone and in combination with anti-programmed cell death protein 1 ligand 1 (αPD-L1) to mice with B16.SIY melanoma results in increased gut *Bifidobacterium* and decreased tumor growth.^133^ The reduction in tumor growth is dependent on *Bifidobacterial*-stimulated DC maturation, which enhances CD8 T cell priming and accumulation in the tumor microenvironment.^133^ *B. breve* strain JCM92 also augments oxaliplatin’s anti-tumor efficacy in MC38 colon carcinoma-bearing mice compared with oxaliplatin alone, with increased intra-tumor CD8 T cells as well as increased CD4/Treg and CD8/Treg ratios.^134^ This effect is also true with *B. breve* JCM92 PD-1 blockade, with increased intra-tumor CD8 T cells and CD8/Treg ratios compared to PD-1 blockade alone. At the transcriptional level, *B. breve* JCM92 results in higher IL-2, STAT5 signaling, and IFN-γ responses compared to mice treated with oxaliplatin or PD-1 blockade and control bacteria. Addition of *B. infantis* to monoclonal antibody and radiotherapy treatment of Lewis lung carcinoma in mice slows tumor growth and prolongs animal survival.^135^ In the same model, *B. pseudolongum* is significantly elevated in the gut of mice with delayed or absent tumorigenesis.^136^

In addition to augmenting anti-cancer therapies, Bifidobacteria has also been shown to have independent anti-tumor activity. *B. breve* lw01 administration suppresses head and neck squamous cell carcinoma growth in mice, mediated by the upregulation of CCL20, which is associated with increased migration of CD11c DCs to ileal villi and tumor microenvironment via upregulation of IL-12^137^. Furthermore, Bifidobacteria ameliorate checkpoint inhibitor-associated autoimmunity. For examples, mice receiving anti-CTLA-4 with superimposed DSS colitis also receiving a cocktail of *Bifidobacterium bifidum, Bifidobacterium longum, Bifidobacterium lactis, and Bifidobacterium breve* show less colitis compared to no bacteria controls without impacting anti-tumor efficacy against B16F10 melanoma.^138^ Tregs are required for this protective effect as it is abrogated in Treg-depleted mice. Despite their broad tolerogenic immune function, Bifidobacteria are also capable of contributing alone or in concert with immunotherapies and chemotherapies to increase host anti-tumor activity.

### Pipeline for discovery

Over the past several years, the use of untargeted metabolomics to detect alterations in systemic metabolism has greatly expanded. This technology has provided a hypothesis generating engine for labs studying the metabolite mediators generated or induced by members of the gut microbiota. Other groups have begun to employ spatial transcriptomics to compare local transcriptional changes in cells at the host microbiota interface.^139^ Application of a spatial omics approaches have proven critical to studying microbiota–gut interface given the significant artifacts that accompany cellular disaggregation protocols used to isolate mucosal cell populations for flow cytometry or single-cell RNA sequencing (scRNAseq). Depending on the protocol used to produce single-cell suspensions for assays, the composition of immune cells from similar starting samples will differ.^140^ However, both tools only demonstrate association rather than causation. More recently, teams have begun to adopt a hybrid spatial metabolomics approach, which permits spatial resolution of metabolic changes in the host.^136^ Authors localized metabolic changes, particularly in glycolysis and amino acid catabolism, in tumor tissue in mice administered Lewis Cancer cells and subsequent *Akkermansia muciniphila* treatment. For example, lactic acid (highly expressed in tumor tissue) was downregulated in the tumors of mice treated with *Akkermansia*, with confirmed downregulation of lactate dehydrogenase-A enzyme via immunofluorescence. Again, as hypothesis generators, this omics pipeline identifies small molecules of interest both for mechanic studies in vitro and in vivo, but also for development of potential diagnostic biomarkers and therapeutic interventions. While these techniques have not been applied directly to the study of *Bifidobacteria*, these early studies provide a blueprint.

## Data Availability

Data sharing is not applicable to this article as no new data were created or analyzed in this work.
